# The Cost of Uncertainty: A Microcosting Analysis of the Cancer of Unknown Primary Diagnostic Pathway

**DOI:** 10.1038/s44276-026-00252-8

**Published:** 2026-10-23

**Authors:** Laura Spurgeon, Claire Mitchell, Alicia-Marie Conway, Natalie Cook, Sean P. Gavan

**Affiliations:** 1 Department of Medical Oncology, The Christie NHS Foundation Trust, Manchester, UK; 2 Division of Cancer Sciences, School of Medical Sciences, Faculty of Biology, Medicine and Health, The University of Manchester, Manchester, UK; 3 National Biomarker Centre, Paterson Building, Manchester, UK; 4 Manchester Centre for Health Economics, Division of Population Health, Health Services Research and Primary Care, School of Health Sciences, Faculty of Biology, Medicine and Health, The University of Manchester, Manchester, UK

## Abstract

**Background:**

Cancer of Unknown Primary (CUP) is a heterogeneous group of cancers for which a primary site cannot be identified. The diagnostic pathway is often protracted and resource-intensive, with little known about the costs involved. The primary aims of this study were to (a) determine time-to-treatment-decision (pathway duration) and (b) estimate resources incurred and direct healthcare costs within the UK’s diagnostic pathway.

**Methods:**

A microcosting analysis was conducted on 370 patients with CUP. For relevant context, a comparison cohort of 216 patients with stage IV cholangiocarcinoma (CCA) was selected. Pathways were mapped from the first cancer-related investigation until treatment decision. Analyses estimated pathway duration and cost, and explored the significance of prognostic variables on the CUP pathway.

**Results:**

Median pathway duration in CUP was 40% longer than CCA (2.8 months vs 2.0 months, respectively, *p* < 0.001). Mean per-patient cost of the CUP pathway was 62% higher than CCA (£3902 vs £2403 respectively, *p* < 0.001). Performance status 0-1 and squamous-cell histology were predictive of higher costs in CUP.

**Conclusions:**

Direct healthcare costs were significantly higher in CUP. Improvements are required to rationalise investigations and facilitate patients receiving treatment in an appropriate timeframe.

## Introduction

Cancer of Unknown Primary (CUP) represents an heterogenous group of metastatic cancers, for which the primary site cannot be identified despite thorough diagnostic work-up. It accounts for around 2% of all new cancer diagnoses in the United Kingdom (UK) each year [1]; however its aggressive nature, together with the paucity of effective treatment options, mean it has a disproportionately high mortality rate [2]. As a result, it remains the sixth leading cause of cancer-related death in the UK [1].

Patients presenting with disseminated cancer undergo extensive investigations in an attempt to determine the tissue-of-origin (TOO) if a primary site is not readily identifiable. For the majority of patients, a primary site will be identified or strongly suspected after appropriate investigations; for the remaining patients, a diagnosis of confirmed CUP is made [3]. Currently, CUP remains a diagnosis of exclusion with patients undergoing myriad investigations based on their sex, pattern of disease, and suspected primary site (Supplementary File 1). Whilst guidelines aim to standardise diagnostic pathways and reduce variability, several additional factors can influence the choice of investigations, such as patient preference; performance status and suitability for treatment; clinician expertise and experience; and access to specialist diagnostic tests, such as whole genome sequencing (WGS), which, although approved by NHS England for patients with confirmed CUP [4], can be challenging due to the need for invasive repeat biopsies [5] and the difficulties associated with obtaining high-quality tumour tissue in CUP [6].

The clinical presentation of CUP can also vary widely, typically determined by the site/s of disease, although many patients report vague, non-specific symptoms which can pose a diagnostic challenge and delay appropriate specialty referrals [7]. Patients with CUP are also more likely to be hospitalised in the months prior to a formal diagnosis, likely due to rapidly progressive disease, a protracted diagnostic pathway, and delayed treatment initiation [8]. Although the pathway is highly variable and time-consuming, impacting both patient experience and clinical outcomes [9], very little is known about the resources and costs involved in determining a diagnosis in current UK practice. However, analyses in Canada and Australia suggest pre-diagnostic costs for CUP are significantly higher than for patients with metastatic cancers of known primary sites [8, 10, 11].

Quantifying healthcare resource usage and associated costs in the CUP pathway is essential for future resource allocation and planning, improving both diagnostic efficiency and patient experience, and providing an important benchmark for evaluating new diagnostic techniques and the wider impact of primary site identification in CUP. The need for this economic analysis has become ever more pertinent with the emergence of new technologies which can predict TOO via tissue- or blood-based genetic analysis, offering the potential to streamline the diagnostic pathway, increase the number of patients receiving site-specific treatment, and improve patient outcomes [12–15]. Microcosting is a valuable methodology to estimate the required resources and costs incurred along highly variable diagnostic or treatment pathways and is widely used in the context of complex diagnostic testing strategies [16, 17]. It uses detailed bottom-up resource utilisation and unit cost data to generate estimates of the economic cost involved with a particular test or treatment [17], and is an appropriate methodology to evaluate the CUP diagnostic pathway.

This study represents the first UK-based microcosting analysis of the CUP diagnostic pathway. Its primary aims were to: (a) determine the time-to-treatment-decision for patients with CUP (pathway duration), and (b) estimate the resources incurred and associated direct healthcare costs within the current pathway. The secondary aims were to: (a) explore how prognostic clinical variables influence both the duration and cost of the CUP diagnostic pathway; (b) estimate the population-level cost of the CUP diagnostic pathway; and (c) determine the time-to-treatment-decision and direct healthcare costs of another hard-to-diagnose cancer for relevant comparison.

## Methods

### Study design

A retrospective cohort comparison study was undertaken and reported according to the STROBE checklist [18]. The microcosting analysis assumed the NHS (service provider) perspective to identify the resource use and per-patient cost of the diagnostic pathways. Direct costs associated with the delivery of the pathway were identified, whilst indirect non-medical costs incurred outside of the healthcare system, such as those from a societal perspective, were not included as they were beyond the scope of the study. The project was approved by the Quality Insurance and Clinical Audit Committee at the tertiary cancer hospital (reference 4582), and was performed in accordance with the Declaration of Helsinki.

### Participants

**Fig. 1:**
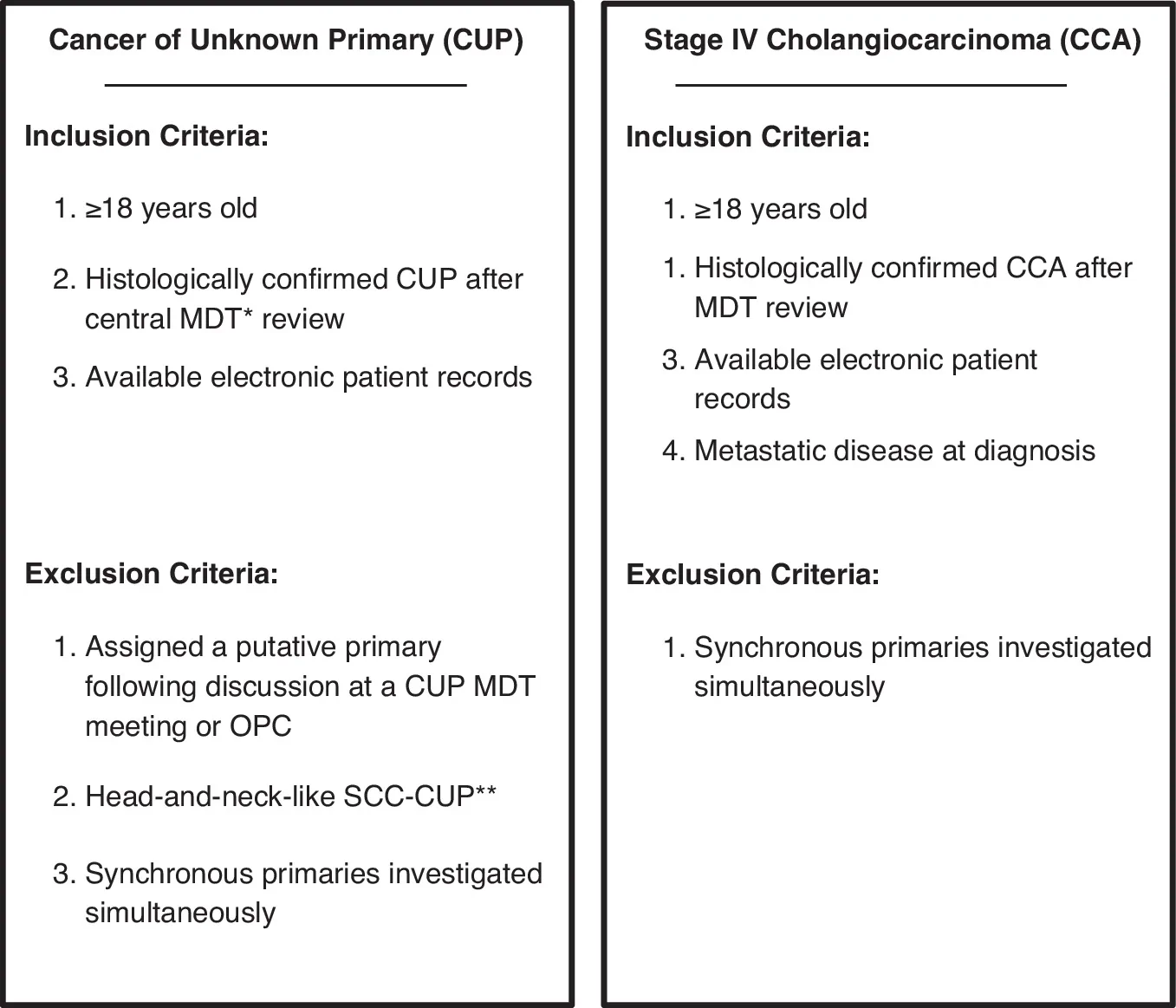
Inclusion and exclusion criteria for CUP and CCA cohorts. *MDT multidisciplinary team, OPC outpatient clinic, SCC squamous cell carcinoma; **excluded as this group of patients typically follows diagnostic and treatment pathways akin to their putative primary.

Costs were determined using real-world data from consecutive patients with histologically confirmed CUP, referred to a tertiary cancer hospital between January 2015 and December 2024. Inclusion and exclusion criteria are detailed in Fig. 1.

A comparison cohort of patients diagnosed with stage IV cholangiocarcinoma (CCA), referred to the same tertiary hospital between January 2021 and December 2025, was selected to provide relevant context to the findings. CCA is a comparably rare, hard-to-diagnose cancer and was an appropriate comparison cohort due to its similarities with CUP, including: advanced-stage presentation due to delayed or absent symptoms; frequent diagnosis following emergency presentation [19]; poor overall survival for advanced disease [19]; biological aggressiveness [20]; absence of a validated screening programme; and diagnostic challenge due to its anatomic location and lack of reliable biomarkers [20, 21].

### Data collection and analysis

#### Stage 1: identifying the pathways and use of resources

Electronic patient records (EPR) from the tertiary cancer hospital were reviewed for the CUP and CCA cohorts. Resource units in each patient’s pathway were mapped from the first cancer-related diagnostic investigation in secondary care until an initial treatment decision. Resource units included in the analysis were secondary care outpatient clinic (OPC) appointments; radiological imaging; diagnostic interventions (e.g. endoscopic procedures); tissue biopsies, including pathology analysis; ascitic or pleural fluid sampling, including cytology analysis; molecular testing used to try and identify a primary site (such as WGS); and multidisciplinary team (MDT) discussions.

The majority of resource units were conducted at local hospitals prior to referral to the tertiary cancer centre. However, at the point of referral, reports from diagnostic investigations, imaging, endoscopic procedures, pathology, cytology, and molecular testing were uploaded onto the EPR system, along with clinical correspondence relating to OPCs and MDT outcomes, allowing them to be reviewed and accurately costed in this analysis.

Resource units excluded from the microcosting analysis due to insufficient access to relevant data included primary care appointments; Accident and Emergency (A&E) attendances; and routine blood-based tumour markers (CA125, CA19-9, αFP, CEA, PSA). Inpatient admissions could also not be included as access to EPRs was restricted to the tertiary cancer hospital only, and the vast majority of patients (91%) who had unplanned inpatient admissions during their diagnostic pathway were admitted to local hospitals, meaning records could not be accessed. Therapeutic interventions without diagnostic utility (e.g. oesophageal/biliary stent insertion or indwelling abdominal catheters); investigations or clinic appointments undertaken in the private setting or as part of a clinical trial; healthcare professionals’ time; and costs associated with prescribed medications were also not included as they were beyond the scope of the study.

Tissue and/or blood-based molecular or pathological testing conducted after a confirmed diagnosis and used purely for treatment stratification was not included in the analysis.

#### Stage 2: identifying unit costs

Costs for each resource unit were identified from the NHS National Cost Collection Database [22] (price year 2023/24), using the national mean unit cost per activity. The most appropriate cost code for each resource unit was chosen based on information from the electronic patient records and/or clinician judgement. If a specific resource unit was not available on the NHS National Cost Collection Database, alternative sources were sought to appropriately cost the resource unit (Supplementary File 2). Costs were calculated in Great British Pounds (GBP).

#### Stage 3: data analysis

##### Cohort validation

Demographic data and disease characteristics were compared using Chi-square (categorical data) and Mann-Whitney U (continuous data). To validate the cohorts, Kaplan-Meier statistics were used to calculate median overall survival (mOS), which was determined from the date of histological diagnosis until death from any cause or the point of data cut-off. To confirm differences in survival outcomes by histological subtype in CUP, mOS was also calculated for undifferentiated carcinoma (UC), adenocarcinoma (AC), and squamous cell carcinoma (SCC), with UC/AC grouped together for subsequent analyses due to similar outcomes [23, 24].

Cox multivariable regression analysis was undertaken to explore the impact of known prognostic clinical variables on OS in both CUP [24] and CCA [19].

##### Pathway duration

Total pathway durations were calculated from the date of first cancer-related investigation in secondary care until the date of an initial treatment decision as a confirmed CUP or CCA. An odds ratio (OR) was calculated to determine the likelihood of receiving an initial treatment decision within 2.0 months (62 days). Logistic regression analysis was used to determine the impact of pathway duration on the likelihood of receiving anti-cancer treatment.

##### Pathway cost

Total and mean per-patient costs were calculated by combining the cost of each resource unit in each patient pathway. Means were considered statistically appropriate to use despite the costing data being non-normally distributed, as the sample sizes were sufficiently large (*n* = 370 and *n* = 216) to meet the requirements for application of the Central Limit Theorem [25].

##### Impact of prognostic clinical variables on Cancer of Unknown Primary pathway

To explore whether any known prognostic clinical variables were influencing the duration or cost of the CUP pathway, subgroup analyses were performed. First, univariable analyses were conducted, using Mann-Whitney U to explore the difference in pathway duration and independent two-sided *t*-tests to explore the difference in pathway cost.

Using the significant prognostic clinical variables identified in the univariable analysis, multivariable linear regression analysis was undertaken for both the duration and cost of the CUP pathway. β-coefficients are provided in raw units for duration (months) and cost (GBP).

To account for the skewed distribution of the data, generalised linear model (GLM) regression analysis (family: gamma; link function: log) was then performed. To interpret the analysis, the coefficient (e^β^) was exponentiated using a percentage calculation to provide a percentage change in the dependent variable based on a 1-unit increase in the independent variable.

##### Pre- and post-2021 subgroup analyses

To confirm the robustness of the findings with respect to the difference in time periods between the CUP and CCA cohorts, subgroup analyses were undertaken in the CUP cohort (pre-2021 vs post-2021) using Kaplan-Meier statistics, Mann-Whitney U and independent *t*-test analyses to determine any difference in OS, pathway duration or mean per-patient cost, respectively.

A p-value of <0.05 was used to determine statistical significance for all analyses. Analyses were performed on IBM SPSS Statistics, version 30.

##### Population level extrapolation for Cancer of Unknown Primary

To extend the cost analysis, a probabilistic model-based simulation was performed to extrapolate the reported per-patient costs to a national level. Total population size in the UK was determined from the Office for National Statistics [26]. Data on the age-standardised incidence rate of CUP per year were obtained from Cancer Research UK statistics for the International Classification of Diseases 10 (ICD-10) codes C77-C80 [4]. Probability distributions (log-normal) were used to characterise uncertainty in the incidence rates, whilst a gamma distribution was used to characterise uncertainty in the per-patient cost, using the estimated mean and standard error from the patient-level data analysis. The calculations were repeated 5000 times using a Monte Carlo simulation to propagate parameter uncertainty. The population-level simulation was performed using Microsoft 365 Excel.

## Results

### Cancer of Unknown Primary

#### Cohort validation

**Fig. 2:**
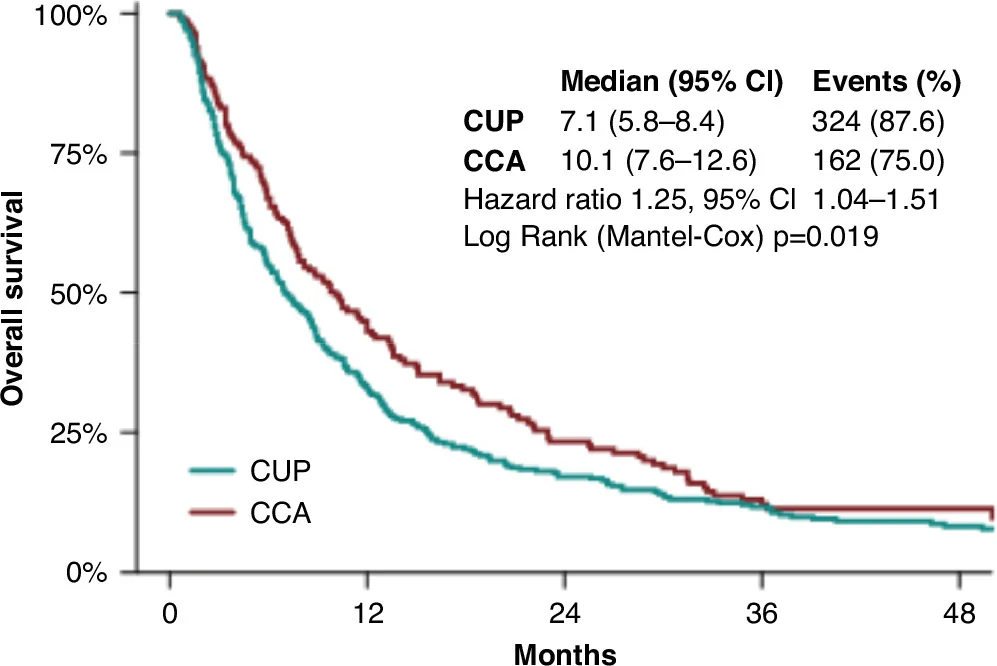
Kaplan-Meier curve for overall survival in CUP and CCA.

Between January 2015 and December 2024, 370 patients with confirmed CUP meeting the eligibility criteria were referred to the tertiary hospital. Demographic details are provided in Table 1. The median age at diagnosis was 65 years (range 25–93), with a mOS of 7.1 months (95% CI 5.8–8.4; 324 events) for the whole cohort (Fig. 2). Eighty-six percent (319/370) of patients had UC or AC histological subtypes, with the remaining 14% SCC. The mOS of UC, AC, and SCC was 6.2 months (95% CI 4.9–7.5; 102 events), 6.2 months (95% CI 4.1–8.3; 189 events), and 16.3 months (95% CI 8.2–24.4; 34 events), respectively.

**Table 1:** Patient demographics and clinical variables for CUP and CCA cohorts.

Patient details	CUP * 370 patients	CCA *216 patients*	*P*-value
Sex			
Male	183 (49.5%)	105 (48.6%)	0.84
Female	187 (51.5%)	111 (51.4%)	
Age at diagnosis in years			
Median (range)	65 (25-93)	66 (19-86)	0.43
Performance status			<0.01
1	194 (52.4%)	145 (67.1%)	
≥2	176 (47.6%)	71 (32.9%)	
Histology			-
UC	116 (31.3%)	-	
AC	203 (54.9%)	-	
SCC	51 (13.8%)	-	
ICCA	-	137 (63.4%)	
PCCA	-	47 (21.2%)	
DCCA	-	31 (14.4%)	
Sites of disease			
Lymph node	208 (56.2%)	115 (53.2%)	<0.01**
Abdomen/pelvis	133 (35.9%)	55 (25.5%)	
Liver	121 (32.7%)	159 (73.6%)	
Lung	112 (30.3%)	38 (17.6%)	
Bone	105 (28.4%)	16 (7.4%)	
Brain	11 (2.9%)	0 (0%)	
Other	8 (2.2%)	0 (0%)	
No. sites of disease***			
<2	282 (76.2%)	100 (46.3%)	<0.01
≥3	88 (23.8%)	116 (53.7%)	

*CUP cancer of unknown primary, *CCA* cholangiocarcinoma, *UC* undifferentiated carcinoma, *AC* adenocarcinoma, *SCC* squamous cell carcinoma, *CCA* cholangiocarcinoma, *ICCA* intrahepatic cholangiocarcinoma, *PCCA* peri-hilar cholangiocarcinoma, *DCCA* distal cholangiocarcinoma, *CI* confidence interval; **Chi-square test for trend; ***includes primary site for CCA cohort.

As expected, Cox multivariable regression analysis found that PS ≥ 2 (hazard ratio [HR] = 2.7, 95% CI 2.1–3.5, *p* < 0.001), UC/AC histology (HR = 1.9, 95% CI 1.3–2.8, *p* = 0.001), presence of liver metastases (HR = 1.3, 95% CI 1.1–1.4), *p* = 0.011) and ≥3 sites of disease (HR = 1.4, 95% CI 1.1–2.0, *p* = 0.015) were poor prognostic clinical variables (Supplementary File 3).

#### Pathway duration

The median time from first investigation in secondary care to initial treatment decision as a confirmed CUP was 2.8 months (range 0.9–13.6; interquartile range 1.9), with 42% (156/370) of patients not receiving any anti-cancer therapy. Only 25% (92/370) of patients received an initial treatment decision within 2.0 months, and 7.3% (27/370) waited ≥6 months.

#### Pathway cost

**Fig. 3:**
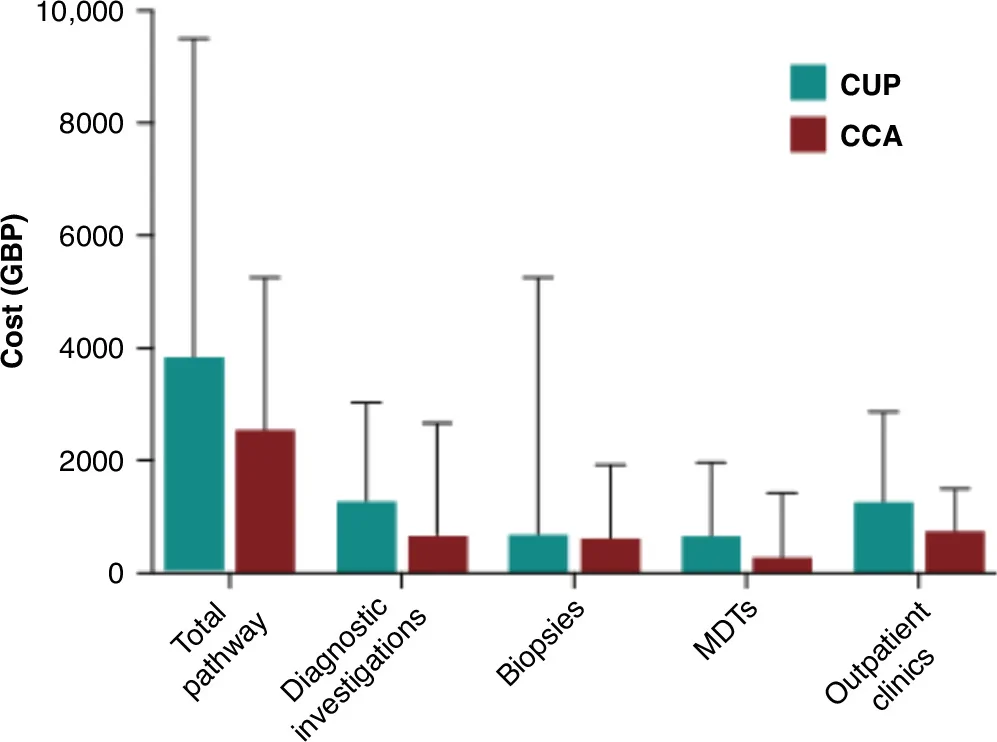
Comparison of resource unit costs in CUP and CCA diagnostic pathways.

The mean per-patient cost of the diagnostic pathway was £3902 (range £1361–9507, 95% CI £3795–4011) (Supplementary File 4). Over 60% of the pathway costs were associated with diagnostic investigations and outpatient clinic appointments, with only 2% spent on molecular testing (Table 2 and Fig. 3). Diagnostic investigations are detailed in Supplementary File 5. The most common imaging modalities were computed tomography [CT] (99.7% of patients), ultrasound scan [USS] (59.7%) and magnetic resonance imaging [MRI] (54.9%), whilst upper gastrointestinal (UGI) endoscopy was the most common endoscopic procedure (42.1%), followed by colonoscopy (21.4%).

**Table 2:** Resource unit allocation and associated costs in the CUP and CCA diagnostic pathways.

	CUP *			CCA		
	Proportion of total pathway cost (%)	Number per patient – mean (range)	Cost in GBP – mean (range)	Proportion of total pathway cost (%)	Number per patient – mean (range)	Cost in GBP – mean (range)
Diagnostic investigations	32.0	5.6 ± 2.0 (2–14)	1274 ± 646 (121–3030)	28.1	3.6 ± 1.5 (1–9)	673 ± 434 (121–2661)
Outpatient clinic appointments	32.0	4.7 ± 1.6 (2–10)	1232 ± 395 (301–2865)	35.0	3.0 ± 0.8 (2–6)	841 ± 216 (301–1538)
Biopsies	17.2	1.3 ± 0.5 (1–3)	678 ± 418 (356–5252)	25.5	1.2 ± 0.5 (1–3)	611 ± 385 (417–1924)
MDT discussions	16.8	4.2 ± 1.4 (2–10)	651 ± 364 (168–1966)	11.4	2.6 ± 0.9 (1–6)	274 ± 175 (95–1418)
Molecular testing	2.0	0.15 ± 0.4 (0–2)	79 ± 224 (0–1000)	-	-	-

*CUP Cancer of Unknown Origin, *CCA* cholangiocarcinoma, *MDT* multidisciplinary team, *GBP* Great British Pounds.

### Impact of prognostic clinical variables on Cancer of Unknown Primary pathway

#### Pathway duration

Univariable Mann-Whitney U analysis was performed to determine whether there was any significant difference in median pathway duration based on prognostic clinical variables. This found that patients with PS 0-1, SCC histology, no liver metastases, <2 sites of disease, and lymph node-only disease had significantly longer pathways (Supplementary File 6).

Multivariable linear regression analysis found PS 0-1 (β = 0.586 (95% CI 0.212–0.9590, *p* = 0.002) and absence of liver metastases (β = 0.531 (95% CI 0.072–0.990, *p* = 0.023) were predictive of significantly longer pathway durations (Table 3). GLM regression analysis confirmed that pathway durations were 20.0% (95% CI 8.7–32.5%, *p* < 0.001) and 19.0% (95% CI 5.3–34.4%, *p* = 0.005) longer for patients with PS 0-1 and no liver metastases, respectively (Table 3).

**Table 3:** Multivariable linear regression and GLM regression analyses of prognostic clinical variables for the CUP pathway.

Variables	Multivariable Linear Regression Analysis				Generalised Linear Model Regression Analysis			
	Pathway Duration		Pathway Cost		Pathway Duration		Pathway Cost	
	β (95% CI) – months	*P value*	β (95% CI) – GBP (£)	*P value*	Exp(β) (95% CI) – % difference	*P value*	Exp(β) (95% CI) – % difference	*P value*
**Performance Status** 0-1 vs ≥2	0.586 (0.212–0.902)	***0.002***	282 (41–522)	***0.022***	20.0 (8.7–32.5)	***<0.001***	7.8 (1.4–14.5)	***0.016***
**Histology** SCC* vs UC/AC	0.357 (−0.188–0.902)	*0.199*	529 (178–880)	***0.003***	10.7 (−4.2–27.9)	*0.166*	13.7 (4.0–24.2)	***0.005***
**Liver Metastases** Absent vs Present	0.531 (0.072–0.990)	***0.023***	202 (−93–498)	*0.179*	19.0 (5.3–34.4)	***0.005***	5.8 (−1.6–13.9)	*0.129*
**Sites of Disease** < 2 vs ≥3	−0.022 (−0.520–0.477)	*0.932*	128 (−193–449)	*0.433*	−1.4 (-13.7–12.5)	*0.831*	3.3 (-4.6–11.9)	*0.426*
**Lymph Node Only** Yes vs No	0.012 (−0.423−0.525)	*0.832*	-	-	15.9 (−9.3-48.2)	*0.237*	-	-

*SCC, squamous cell carcinoma, *UC* undifferentiated carcinoma, *AC* adenocarcinoma; *Exp(β)* exponentiated β, *CI* confidence interval.

Finally, logistic regression analysis was performed to determine whether the duration of the diagnostic pathway influenced the likelihood of receiving any anti-cancer treatment. Interestingly, this showed that longer pathway durations were associated with an increased likelihood of receiving treatment (odds ratio [OR] = 1.3, 95% CI 1.1–1.5, *p* < 0.001). Patients with a pathway duration of ≥2.8 months were more likely to receive radical surgery or radiotherapy than patients with pathway durations of <2.8 months.

#### Pathway cost

Univariable independent *t*-test analysis found costs were significantly higher for patients who were PS 0-1 vs PS ≥ 2, SCC vs UC/AC histological subtype, no liver metastases vs liver metastases, and <2 vs ≥3 sites of disease (Supplementary File 7). Increased costs for these patients were primarily due to diagnostic investigations and outpatient clinic appointments (Supplementary File 8).

Multivariable linear regression analysis found PS 0-1 (β = 282 (95% CI 41–522, *p* = 0.022) and SCC histology (β = 529 (95% CI 178–880, *p* = 0.003) were predictive of significantly more expensive pathways (Table 3). GLM regression analysis showed that pathway costs were 7.8% (95% CI 1.4–14.5%, *p* = 0.016) and 13.7% (95% CI 4.0–24.2%, *p* = 0.005) higher for patients with PS 0-1 and SCC histology, respectively (Table 3).

### Pre- and post-2021 subgroup analyses

There was no significant difference in mOS pre-2021 (6.9 months, 95% CI 5.3–8.5) versus post-2021 (7.6 months, 95% CI 5.4–9.7, *p* = 0.963). Mann-Whitney U analysis also found there was no significant difference in median pathway duration pre- and post-2021 (2.7 months (range 0.9–13.6) vs 2.7 months (range 0.9–11.8), respectively, *p* = 0.102). Independent *t*-test analysis confirmed there was no significant difference in mean per-patient costs pre- and post-2021 (£3866 ± 1131 vs £3961 ± 1271 respectively, *p* = 0.454).

### Population level extrapolation for Cancer of Unknown Primary

8100 new cases of CUP are diagnosed every year in the UK, with a projected increase to 8200 by 2038–2040 [1]. Based on the current incidence rate, the annual cost of the CUP diagnostic pathway in the UK is £34.9 million (95% credible interval: £33.7 million - £36 million).

### Stage IV Cholangiocarcinoma

#### Cohort validation

Between January 2021 and December 2025, 216 patients with confirmed stage IV CCA meeting the eligibility criteria were referred to The Christie Hospital (Fig. 1). The median age at diagnosis was 66 years (range 19–86), with a mOS of 10.1 months (95% CI 7.6–12.6; 162 events). Cox multivariable regression analysis showed that PS ≥ 2 (HR = 2.7, 95 CI% 2.0–3.8, *p* < 0.001) was a poor prognostic risk factor; however, age was not prognostic (HR = 0.8, 95% CI 0.7–1.3, *p* = 0.842).

#### Pathway duration

The median time from first investigation in secondary care to initial treatment decision as a confirmed CCA was 2.0 months (range 0.5-6.9; interquartile range 1.7), making the median pathway duration for CUP 40% longer. Over 70% of patients with CCA received anti-cancer therapy (153/216), with >50% (117/216) receiving an initial treatment decision within 2.0 months and 1.4% (3/216) waiting ≥6 months. Patients with a diagnosis of CCA were significantly more likely to receive a treatment decision within 2.0 months (62 days) than patients with CUP (OR = 3.6, 95% CI 2.5-5.1, *p* < 0.001).

#### Pathway cost

The mean per-patient cost of the CCA diagnostic pathway was £2403 (range £1117–5257, 95% CI £2262–2545), making CUP ~ 60% more expensive. The majority of pathway costs were associated with outpatient clinic appointments (35.0%), diagnostic investigations (28.1%), and biopsies (25.5%) (Table 2 and Fig. 3). The most common imaging modalities were CT (100% of patients), MRI (60.2%) and USS (51.4%), whilst endoscopic retrograde cholangiopancreatography [ERCP] (35.6%) and UGI endoscopy (16.7%) were the most common endoscopic procedures (Supplementary File 9).

## Discussion

This is the first microcosting study in the UK to define the duration and direct healthcare costs associated with the CUP diagnostic pathway. We found that to diagnose a patient with CUP costs nearly £4000 on average and takes almost 3 months of diagnostic work-up. This was 1.6 times more expensive than a comparable hard-to-diagnose tumour type, and importantly, patients with stage IV CCA were 3.6 times more likely to receive a treatment decision within 2.0 months (62 days). Patients with CUP used a wider range of healthcare resources, reflective of the extensive diagnostic work-up and heterogeneous presentation of the disease.

Our study provides unique insight into the healthcare resource usage and associated costs involved in diagnosing CUP in the UK, as well as supporting findings from international studies [8, 10, 11]. In a prospective Australian cohort study, Gordon et al. (2023) reported that pre-diagnosis costs in CUP were nearly triple those for ovarian cancer, predominantly due to consultations, imaging, pathology, and investigative procedures [11]. Similarly, Hannouf et al. (2018) found that pre-diagnostic resource costs in Canada were significantly higher in patients with CUP than those with a known primary site [10]. Finally, in another Australian study, Vajdic et al. (2015) used a population-based nested case-control to compare healthcare utilisation in the three months pre- and one month post-diagnosis for patients with CUP and a range of other metastatic cancers. The authors reported wide variability within the CUP diagnostic pathway and in the nature of the diagnostic investigations between CUP and other cancers, with the use of multiple imaging modalities more common in CUP [8].

The median time from first investigation in secondary care to initial treatment decision as a confirmed CUP was 2.8 months, which is non-compliant with the NHS’ 62-day (2.0 month) target for time from referral to treatment initiation [27], with evidence suggesting that every four-week delay in receiving treatment reduces survival by ~10 %[28]. Whilst our study found that longer pathway durations were associated with an increased likelihood of receiving anti-cancer therapy and that patients suitable for active management were being appropriately investigated, delayed treatment initiation in CUP has been shown to have a detrimental impact on outcomes [29]. Furthermore, previous research has shown that delayed treatment initiation is also associated with significantly higher pre-treatment costs in CUP than other metastatic cancers [30], as well as contributing to the anxiety, frustration and uncertainty widely reported in this patient group [31, 32]. Our findings suggest that the time taken to receive a treatment decision in CUP is too long and exceeds national guidelines [27]. Attempting to improve outcomes through identification of the TOO may in fact be negatively impacting patients due to the protracted nature of the current pathway. Urgent improvements are needed to streamline the pathway, rationalise investigations and provide a more accurate diagnosis, potentially through the implementation of TOO classifiers into standard-of-care practice.

Direct pathway costs for CUP were significantly higher than for CCA, and whilst it was expected that patients with CUP would undergo more diagnostic investigations, the inability to identify a primary site in any of our cohort despite their extensive work-up raises important questions about the cost-effectiveness of the current pathway. Furthermore, patients with CUP had significantly more MDT discussions and outpatient appointments, suggesting the resource- and financial-burden of the pathway goes beyond diagnostic tests alone. Given the lack of microcosting studies in the UK, cost comparisons between cancer types are challenging; however, our findings align with similar international analyses [8, 10, 11, 33]. Interestingly, several studies have detailed the cost of more common cancers to the wider economy [34–36], and a recent Cancer Research UK report found that CUP was the 6^th^ leading cause of productivity loss due to cancer-related death [37]. This suggests its impact from both the service provider and societal perspective may be disproportionately high and certainly warrants investigation in future economic evaluations.

Over two-thirds of the CUP pathway costs were associated with diagnostic investigations and outpatient appointments, whilst only 2% was spent on molecular testing. Although WGS has been approved for use in CUP since 2022 [4] and offers the potential to identify a TOO, only three out of 88 (3.4%) eligible patients in our cohort underwent testing outside of a clinical trial, and two analyses failed due to inadequate tissue. Although only a single-site study, it does suggest that WGS is an under-utilised test in this population, possibly due to operational challenges or difficulties in obtaining adequate tissue biopsies [6, 38]. WGS requires a high-quality fresh-frozen sample and cannot be performed on formalin-fixed paraffin-embedded tissue (FFPE) – the standard method for storing tissue samples – with the need for a repeat biopsy contributing to the challenge of performing WGS in this patient cohort. To overcome many of the problems associated with tissue analysis in CUP [6] and to improve primary site identification, several blood-based TOO classifiers have been developed [6]. These assays could offer meaningful improvements if implemented at an early enough stage in the diagnostic pathway, potentially reducing the number of investigations, outpatient appointments and MDT discussions; eliminating the need for a fresh tissue biopsy in order to perform WGS [5], expediting a precise diagnosis; and offering significant clinical benefit [6, 12–15]. Furthermore, previous research has shown that identifying a primary site in CUP may also elicit financial benefits through more efficient allocation of healthcare resources [10]. Thorough clinical and economic evaluation is required prior to the integration of blood-based TOO classifiers into standard-of-care practice; however, our study now provides an important benchmark for this analysis.

We found considerable variability in the healthcare resource usage and associated costs within the CUP cohort. Given the inherent heterogeneity and number of factors that can influence the choice of diagnostic investigations [3, 11], some degree of variability is to be expected. Previous research has shown that age is a key determinant of the extent and nature of diagnostic investigations [39]; however, we found that age itself did not influence either the cost or duration of the CUP pathway. Instead, PS, histological subtype and absence of liver metastases were key factors. Suitability for active treatment is likely to drive this finding, with patients with good prognostic factors being more extensively investigated in an attempt to identify the primary tumour, thereby facilitating access to site-specific therapy and improving outcomes [13]. Diagnostic investigations may be curtailed in those patients with poorer PS or liver metastases due to the need to initiate treatment at an earlier point, and in those who are considered unfit for treatment, regardless of whether the primary site could be identified with further tests. Interestingly, costs were significantly higher for SCC-CUP; this may be due to the higher proportion of patients in this histological subtype with lymph node-only or oligometastatic disease who often have more expensive investigations, such as PET scans, prior to radical therapy [24]. The propensity for SCC-CUP to present with regionalised lymph-node-only disease, which typically follows a more indolent course and can be treated with curative intent by local treatment modalities, also explains why its survival outcomes are superior to other CUP histological subtypes [23].

One of the key strengths of our study was the use of real-world data from large, representative cohorts in a public healthcare system. We have provided relevant context to the cost estimates with an appropriately selected comparison group; CCA is a hard-to-diagnose cancer with similarly poor outcomes to CUP and may represent a more apposite comparator group than other cancer types, such as ovarian [11]. Our study has demonstrated how resources and costs are allocated throughout the pathway, offering pertinent information to local healthcare providers for service planning and delivery costs. Undertaking a precise evaluation of the diagnostic pathway has also provided a framework for future economic assessments in both CUP and other types of cancer, helping to reduce the evidence gap and elicit important information regarding how resource allocation and associated costs vary by cancer type.

There are several limitations with our study. Firstly, it was a single-site study at a specialist cancer centre, and analyses from other sites in the UK are needed to validate our findings. Furthermore, the study was retrospective in nature, which contributed to missing data from non-diagnostic therapeutic procedures, primary care consultations, A&E attendances and inpatient admissions, all of which are likely to contribute costs, meaning we may have underestimated the economic burden of the CUP pathway. Additionally, we have undertaken the analysis from the service provider’s perspective only, meaning wider societal costs have not been considered. There was a time difference between the CUP (2015–2024) and CCA (2021–2025) cohorts, which could have influenced the nature of diagnostic investigations available and associated costs, particularly given the impact of the COVID-19 pandemic on healthcare access between 2020 and 2022. However, our subgroup analysis has shown there was no significant difference in the CUP pathway duration or cost pre- and post-2021, suggesting the time difference was unlikely to have biased the results. Finally, whilst CCA was an appropriate comparator group, the number of patients with intrahepatic CCA was over-represented in our cohort relative to its incidence amongst CCA subtypes, which could have influenced the nature of investigations performed, and there is evidence to suggest CCA can be misclassified as CUP in ~30% of cases [40]; however, all patients in our CUP cohort received specialist radiology and pathology review and were deemed to be ‘true’ CUPs, making it unlikely that our cohort contained a significant number of misclassified CCAs.

In conclusion, our study has shown that both the time-to-treatment-decision and direct healthcare costs associated with the diagnostic pathway are significantly greater for CUP than CCA, despite CCA also being a rare, hard-to-diagnose cancer. The CUP pathway is resource-intensive, time-consuming and costly, whilst also disadvantaging patients and impacting outcomes. Pathway improvements are required to rationalise investigations, expedite a precise diagnosis, and increase the number of patients receiving treatment in an appropriate timeframe.

## Supplementary information

20.05 REVISED supplementary files BJC reports

## Data Availability

The datasets used and analysed during the current study are available from the corresponding author on reasonable request.
