# Supplementary material for: The Cost of Uncertainty: A Microcosting Analysis of the Cancer of Unknown Primary Diagnostic Pathway
Source: BJC Rep. 2026 Oct 23;4:49. doi: 10.1038/s44276-026-00252-8 (PMC13694267; doi:10.1038/s44276-026-00252-8)
Supplement: Supplementary file 1 [file 44276_2026_252_MOESM1_ESM.docx]

**SUPPLEMENTARY FILES**

**Contents**

1. **Supplementary File 1:** Diagnostic work-up for patients with suspected Cancer of Unknown Primary (adapted from NICE Guidelines, 2025).
2. **Supplementary File 2:** Resource units and associated costs used in microcosting analysis.
3. **Supplementary File 3:** Cox multivariable regression analysis of overall survival based on prognostic clinical risk factors in CUP.
4. **Supplementary File 4:** Distribution of per-patient costs (GBP) for the CUP diagnostic pathway.
5. **Supplementary File 5:** Diagnostic investigations performed in CUP diagnostic pathway.
6. **Supplementary File 6:** Mann-Whitney U analysis for CUP pathway duration (months) based on prognostic clinical variables.
7. **Supplementary File 7:** Independent *t*-test analysis for the CUP diagnostic pathway costs (GBP) based on clinical variables.
8. **Supplementary File 8:** Independen*t t*-test analysis for resource units in the CUP diagnostic pathway based on prognostic clinical variables.
9. **Supplementary File 9:** Diagnostic investigations performed in CCA diagnostic pathway.

**Supplementary File 1:** Diagnostic work-up for patients with suspected Cancer of Unknown Primary (adapted from NICE Guidelines, 2025).

| Assessment |  | | Patient Population |
| --- | --- | --- | --- |
| Initial Diagnostic Phase | | | |
| Thorough medical history and physical examination | | | All |
| Blood counts including blood differential test  Biochemistry analyses (including ALP*, LDH, calcium and albumin) | | | All  All |
| Chest X-ray | | | All |
| IV contrast CT thorax, abdomen and pelvis | | | All |
| Tissues biopsy and standard histological examination | | | All |
| Myeloma screen | | | Single or multiple lytic bone lesions |
| Endoscopy | | | Symptom-guided |
| Testicular ultrasound scan | | | Suspected germ cell tumour |
| Second Diagnostic Phase – Special Investigations | | | |
| Tumour markers   1. PSA 2. AFP, β-hCG 3. AFP 4. CA125 | | | 1. Males with suspected prostate cancer 2. Males with suspected germ cell tumour 3. Suspected hepatocellular carcinoma 4. Females with suspected gynaecological primary |
| Upper or lower endoscopy | | | Suspected gastrointestinal primary |
| PET scan | | | 1. Cervical lymphadenopathy with no primary tumour identified on ENT panendoscopy 2. Extra-cervical presentations after discussion at CUP MDT |
| Mammography | | | Suspected breast cancer |
| Breast MRI | | | Adenocarcinoma with axillary involvement after discussion at breast MDT |
| Bronchoscopy | | | Intrapulmonary nodules without evidence of endobronchial disease |
| Molecular Diagnostic Tests | | | |
| IHC antibodies | | All (choice of antibody panels guided by clinical picture) | |
| Whole genome sequencing (tissue) | | All patients with confirmed CUP diagnosis and fresh tissue biopsy | |

*Key: *ALP, alkaline phosphatase; LDH, lactase dehydrogenase; IV, intravenous; CT, computed tomography; PSA, prostate specific antigen; AFP, alpha fetoprotein; β-hCG, βeta-human chorionic gonadotrophin; CA125, cancer antigen 125; PET, positron emission tomography; ENT, ear, nose and throat; MDT, multidisciplinary team; MRI, magnetic resonance imaging; IHC, immunohistochemistry.*

**Supplementary File 2:** Resource units and associated costs used in microcosting analysis.

| Resource Unit | Code | Cost (£) | Source |
| --- | --- | --- | --- |
| Diagnostic colonoscopy with biopsy | 104/FE31Z | 286 | NHS National Cost Collection Database 2023/24 |
| Diagnostic flexible sigmoidoscopy with biopsy | 104/FE34Z | 335 | NHS National Cost Collection Database 2023/24 |
| Diagnostic upper gastrointestinal tract endoscopy with biopsy | 301/FE21Z | 980 | NHS National Cost Collection Database 2023/24 |
| CT* colonoscopy | SI_Imag_OP/ RD61Z | 126 | NHS National Cost Collection Database 2023/24 |
| Plain film x-ray | 812/PF | 42 | NHS National Cost Collection Database 2023/24 |
| CT scan with contrast, 1 area | 812/RD22Z | 126 | NHS National Cost Collection Database 2023/24 |
| CT scan with contrast, 3 areas | 812/RD26Z | 121 | NHS National Cost Collection Database 2023/24 |
| Ultrasound scan without contrast, <20 minutes | SI_Imag_OP/ RD40Z | 61 | NHS National Cost Collection Database 2023/24 |
| MRI scan with contrast, 1 area | 812/RD03Z | 233 | NHS National Cost Collection Database 2023/24 |
| MRI scan with contrast, 3 areas | 812/RD05Z | 225 | NHS National Cost Collection Database 2023/24 |
| PET scan, whole body | 812/RN08A | 384 | NHS National Cost Collection Database 2023/24 |
| Nuclear bone scan | SI_Imag_OP/  RN15A | 509 | NHS National Cost Collection Database 2023/24 |
| Mammogram | N/A | 57 | Hill et al (2023)^(1)^ |
| Transvaginal ultrasound scan | 202/MA36Z | 251 | NHS National Cost Collection Database 2023/24 |
| Diagnostic hysteroscopy with biopsy | 502/MA32Z | 370 | NHS National Cost Collection Database 2023/24 |
| Diagnostic cystoscopy | 101/LB72A | 258 | NHS National Cost Collection Database 2023/24 |
| Diagnostic bronchoscopy | 340/DZ69A | 1077 | NHS National Cost Collection Database 2023/24 |
| Diagnostic nasopharyngoscopy | 120/CA17A | 233 | NHS National Cost Collection Database 2023/24 |
| Diagnostic ERCP | 105/GB11Z | 414 | NHS National Cost Collection Database 2023/24 |
| EUS of hepatobiliary or pancreatic duct | 105/GB13Z | 1635 | NHS National Cost Collection Database 2023/24 |
| Wireless capsule endoscopy | 301/FE50A | 194 | NHS National Cost Collection Database 2023/24 |
| EBUS | 136/DZ70Z | 899 | NHS National Cost Collection Database 2023/24 |
| Percutaneous punch biopsy of the liver | 811/YG11A | 214 | NHS National Cost Collection Database 2023/24 |
| Neck lymph node biopsy | 811/YC01Z | 309 | NHS National Cost Collection Database 2023/24 |
| Chest wall lesion or pleura biopsy | 811/YD02Z | 435 | NHS National Cost Collection Database 2023/24 |
| Lung or mediastinum biopsy | 811/YD03Z | 187 | NHS National Cost Collection Database 2023/24 |
| Abdominal cavity biopsy | 811/YF05Z | 294 | NHS National Cost Collection Database 2023/24 |
| Bone biopsy | 811/YH31A | 242 | NHS National Cost Collection Database 2023/24 |
| Axillary lymph node biopsy | 811/YJ04Z | 249 | NHS National Cost Collection Database 2023/24 |
| Kidney biopsy | 811/YL20A | 76 | NHS National Cost Collection Database 2023/24 |
| Bone marrow biopsy | 303/SA33Z | 452 | NHS National Cost Collection Database 2023/24 |
| Muscle or connective tissue biopsy | 110/YH32A | 263 | NHS National Cost Collection Database 2023/24 |
| Breast biopsy | 811/YJ13Z | 356 | NHS National Cost Collection Database 2023/24 |
| Minimal intracranial procedure (brain biopsy) | 150/AA57A | 359 | NHS National Cost Collection Database 2023/24 |
| Pleural fluid aspiration | 811/YD05Z | 280 | NHS National Cost Collection Database 2023/24 |
| Cytology | 822/PATH01 | 46 | NHS National Cost Collection Database 2023/24 |
| Histology and pathology | 370/PATH02 | 203 | NHS National Cost Collection Database 2023/24 |
| Whole genome sequencing (tissue) | N/A | 1000 | Roche (expert opinion) |
| NGS panel | N/A | 651 | NICE^(2)^ |
| Breast MDT | 370/CMDTB | 86 | NHS National Cost Collection Database 2023/24 |
| Lower gastrointestinal MDT | 370/CMDTB | 126 | NHS National Cost Collection Database 2023/24 |
| Upper gastrointestinal MDT | 370/CMDTB | 95 | NHS National Cost Collection Database 2023/24 |
| Lung MDT | 340/CMDTB | 441 | NHS National Cost Collection Database 2023/24 |
| Gynaecology MDT | 370/CMDTB | 119 | NHS National Cost Collection Database 2023/24 |
| Urology MDT | 101/CMDTB | 228 | NHS National Cost Collection Database 2023/24 |
| Haematology MDT | 303/CMDTB | 186 | NHS National Cost Collection Database 2023/24 |
| Head and neck/ENT MDT | 120/CMDTB | 188 | NHS National Cost Collection Database 2023/24 |
| Neuro-oncology MDT | 150/CMDTB | 296 | NHS National Cost Collection Database 2023/24 |
| Other medical oncology MDT | 370/CMDTB | 93 | NHS National Cost Collection Database 2023/24 |
| Other cancer MDT | 999/CMDTB | 75 | NHS National Cost Collection Database 2023/24 |
| Consultant-led colorectal OPC, F2F (new) | 104/WF01B | 191 | NHS National Cost Collection Database 2023/24 |
| Consultant-led colorectal OPC, F2F (follow up) | 104/WF01A | 149 | NHS National Cost Collection Database 2023/24 |
| Consultant-led hepatobiliary OPC, F2F (new) | 105/WF01B | 279 | NHS National Cost Collection Database 2023/24 |
| Consultant-led hepatobiliary OPC, F2F (follow up) | 105/WF01A | 316 | NHS National Cost Collection Database 2023/24 |
| Consultant-led urology OPC, F2F (new) | 101/WF01B | 190 | NHS National Cost Collection Database 2023/24 |
| Consultant-led urology OPC, F2F (follow up) | 101/WF01A | 163 | NHS National Cost Collection Database 2023/24 |
| Consultant-led spinal surgery OPC, F2F (new) | 108/WF01B | 239 | NHS National Cost Collection Database 2023/24 |
| Consultant-led spinal surgery OPC, F2F (follow up) | 108/WF01A | 204 | NHS National Cost Collection Database 2023/24 |
| Consultant-led neurosurgical OPC, F2F (new) | 150/WF10B | 314 | NHS National Cost Collection Database 2023/24 |
| Consultant-led neurosurgical OPC, F2F (follow-up) | 150/WF10A | 244 | NHS National Cost Collection Database 2023/24 |
| Consultant-led ENT OPC, F2F (new) | 120/WF01B | 185 | NHS National Cost Collection Database 2023/24 |
| Consultant-led ENT OPC, F2F (follow up) | 120/WF01A | 163 | NHS National Cost Collection Database 2023/24 |
| Consultant-led gastroenterology OPC, F2F (new) | 301/WF10B | 238 | NHS National Cost Collection Database 2023/24 |
| Consultant-led gastroenterology OPC, F2F (follow up) | 301/WF10A | 193 | NHS National Cost Collection Database 2023/24 |
| Consultant-led respiratory OPC, F2F (new) | 340/WF10B | 278 | NHS National Cost Collection Database 2023/24 |
| Consultant-led respiratory OPC, F2F (follow up) | 340/WF10A | 203 | NHS National Cost Collection Database 2023/24 |
| Consultant-led gynaecology OPC, F2F (new) | 503/WF10B | 222 | NHS National Cost Collection Database 2023/24 |
| Consultant-led gynaecology OPC, F2F (follow up) | 503/WF10A | 191 | NHS National Cost Collection Database 2023/24 |
| Consultant-led haematology OPC, F2F (new) | 303/WF10B | 314 | NHS National Cost Collection Database 2023/24 |
| Consultant-led haematology OPC, F2F (follow up) | 303/WF10A | 204 | NHS National Cost Collection Database 2023/24 |
| Consultant-led trauma and orthopaedics OPC, F2F (new) | 110/WF10B | 192 | NHS National Cost Collection Database 2023/24 |
| Consultant-led trauma and orthopaedics OPC, F2F (follow up) | 110/WF1A | 168 | NHS National Cost Collection Database 2023/24 |
| Consultant-led dermatology OPC, F2F (new) | 330/WF10B | 177 | NHS National Cost Collection Database 2023/24 |
| Consultant-led dermatology OPC, F2F (follow up) | 330/WF10A | 168 | NHS National Cost Collection Database 2023/24 |
| Consultant-led clinical oncology OPC, F2F (new) | 800/WF10B | 248 | NHS National Cost Collection Database 2023/24 |
| Consultant-led clinical oncology OPC, F2F (follow up) | 800/WF10A | 157 | NHS National Cost Collection Database 2023/24 |
| Consultant-led medical oncology OPC, F2F (new) | 370/WF10B | 301 | NHS National Cost Collection Database 2023/24 |
| Consultant-led medical oncology OPC, F2F (follow up) | 370/WF10A | 193 | NHS National Cost Collection Database 2023/24 |

*Key: *CT, computed tomography; MRI, magnetic resonance imaging; PET, positron emission tomography; ERCP, endoscopic retrograde cholangiopancreatography; EUS, endoscopic ultrasound; EBUS, endobronchial ultrasound; NGS, next generation sequencing; OPC, outpatient clinic; F2F, face-to-face.*

**Supplementary File 3:** Cox multivariable regression analysis of overall survival based on prognostic clinical risk factors in CUP.

|  | Cox regression analysis | |
| --- | --- | --- |
|  | **Hazard ratio (95% CI)** | ***P value*** |
| Patient Age  ≥65 years | 0.8 (0.6-1.1) | *0.113* |
| Performance Status  ≥2 | 2.7 (2.1-3.5) | ***<0.001*** |
| Histology  UC/AC * | 1.9 (1.3-2.8) | ***0.001*** |
| Liver Metastases  Present | 1.3 (1.1-1.4) | ***0.011*** |
| Sites of Disease  ≥3 | 1.4 (1.1-2.0) | ***0.015*** |
| Lymph Node Only  No | 1.7 (0.8-3.3) | *0.114* |

*Key: *UC, undifferentiated carcinoma; AC, adenocarcinoma; CI, confidence interval.*

**Supplementary File 4:** Distribution of per-patient costs (GBP) for the CUP diagnostic pathway.

**Supplementary File 5:** Diagnostic investigations and molecular testing performed in CUP diagnostic pathway.

| Diagnostic Investigation | Number of Patients (%) n=370 |
| --- | --- |
| Imaging  CT*  Ultrasound  MRI  PET  Plain film x-ray  Mammogram  Nuclear bone scan  CT colonoscopy | 369 (99.7%)  231 (62.4%)  203 (54.9%)  197 (53.2%)  181 (48.9%)  34 (9.2%)  28 (7.6%)  8 (2.2%) |
| Endoscopic Procedures  Upper GI endoscopy  Colonoscopy  Cystoscopy  ERCP  Hysteroscopy  Flexible sigmoidoscopy  Nasendoscopy  EBUS  Bronchoscopy  Wireless endoscopy  EUS | 156 (42.2%)  79 (21.4%)  20 (5.4%)  13 (3.5%)  12 (3.2%)  12 (3.2%)  11 (2.9%)  11 (2.9%)  8 (2.2%)  2 (0.5%)  1 (0.3%) |
| Molecular Testing  WGS (88/370 patients eligible**) | 3 (3.4%) |

*Key: *CT, computed tomography; MRI, magnetic resonance imaging; PET, position emission tomography; GI, gastrointestinal; ERCP, endoscopic retrograde cholangiopancreatography; EBUS, endoscopic bronchial ultrasound; EUS, endoscopic ultrasound; WGS, whole genome sequencing; **based on introduction of WGS to standard-of-care practice for patients with CUP in November 2022.*

**Supplementary File 6:** Mann-Whitney U analysis for CUP pathway duration (months) based on prognostic clinical variables.

|  | Duration in months | Mann-Whitney U | |
| --- | --- | --- | --- |
|  | **Median (range)** | **Z score** | ***P value*** |
| Patient Age  <65 years  ≥65 years | 2.7 (0.9-13.6)  2.8 (0.9-11.8) | -1.38 | *0.168* |
| Performance Status  0-1  ≥2 | 3.2 (0.9-13.6)  2.6 (0.9-9.4) | -3.78 | ***<0.001*** |
| Histology  SCC *  UC/AC | 3.3 (1.1-8.9)  2.7 (0.9-13.6) | -2.17 | ***0.03*** |
| Liver Metastases  Absent  Present | 3.3 (0.9-13.6)  2.8 (0.9-9.4) | -3.18 | ***0.001*** |
| Sites of Disease  <2  ≥3 | 2.9 (0.9-11.8)  2.7 (1.3-13.6) | -1.99 | ***0.046*** |
| Lymph Node Only  Yes  No | 3.1 (0.9-11.8)  2.8 (0.9-13.6) | -2.19 | ***0.028*** |

*Key: *SCC, squamous cell carcinoma; UC, undifferentiated carcinoma; AC, adenocarcinoma.*

**Supplementary File 7:** Independent *t*-test analysis for the CUP diagnostic pathway costs (GBP) based on clinical variables.

|  | Cost (GBP) * | |
| --- | --- | --- |
|  | **Mean ± SD** | ***P value*** |
| Patient Age  <65 years  ≥65 years | £3977 ± 1266  £3828 ± 1143 | *0.227* |
| Performance Status  0-1  ≥2 | £4080 ± 1236  £3706 ± 1099 | ***0.002*** |
| Histology  SCC  UC/AC | £4462 ± 1336  £3813 ± 1137 | ***<0.001*** |
| Liver Metastases  Absent  Present | £4026 ± 1225  £3649 ± 1062 | ***0.004*** |
| Sites of Disease  <2  ≥3 | £3977 ± 1176  £3661 ± 1194 | ***0.029*** |
| Lymph Node Only  Yes  No | £4002 ± 1325  £3872 ± 1140 | *0.367* |

*Key: *GBP, Great British pounds; SCC, squamous cell carcinoma; UC, undifferentiated carcinoma; AC, adenocarcinoma.*

**Supplementary File 8:** Independent *t*-test analysis of mean resource units in the CUP diagnostic pathway based on clinical variables.

|  | Dx Ix * | | Biopsy | | MDT | | OPC | |
| --- | --- | --- | --- | --- | --- | --- | --- | --- |
|  | **Mean ±SD** | ***P value*** | **Mean ±SD** | ***P value*** | **Mean ±SD** | ***P value*** | **Mean ±SD** | ***P value*** |
| Patient Age  <65 years  ≥65 years | 5.9 ± 2.1  5.4 ± 1.9 | ***0.025*** | 1.3 ± 0.5  1.2 ± 0.5 | *0.069* | 4.3 ± 1.5  4.1 ± 1.3 | *0.439* | 4.7 ± 1.6  4.8 ± 1.5 | *0.521* |
| Performance Status  0-1  ≥2 | 5.8 ± 2.0  5.4 ± 2.0 | ***0.044*** | 1.3 ± 0.5  1.3 ± 0.5 | *0.687* | 4.3 ± 1.4  4.1 ± 1.3 | *0.314* | 4.9 ± 1.6  4.5 ± 1.5 | ***0.021*** |
| Histology  SCC  UC/AC | 6.2 ± 2.0  5.6 ± 1.9 | ***0.033*** | 1.3 ± 0.5  1.3 ± 0.5 | *0.875* | 4.9 ± 1.6  4.1 ± 1.3 | ***<0.001*** | 6.0 ± 1.4  4.5 ± 1.5 | ***<0.001*** |
| Liver Metastases  Absent  Present | 5.8 ± 2.1  5.3 ± 1.8 | ***0.021*** | 1.3 ± 0.5  1.2 ± 0.4 | ***0.012*** | 4.4 ± 1.4  3.9 ± 1.3 | ***<0.001*** | 4.8 ± 1.7  4.5 ± 1.4 | *0.062* |
| Sites of Disease  <2  ≥3 | 5.7 ± 2.0  5.5 ± 2.0 | *0.452* | 1.3 ± 0.5  1.3 ± 0.5 | *0.995* | 4.3 ± 1.3  4.0 ± 1.5 | *0.072* | 4.8 ± 1.6  4.4 ± 1.5 | *0.053* |
| Lymph Node Only  Yes  No | 5.5 ± 2.3  5.7 ± 1.9 | *0.139* | 1.3 ± 0.5  1.3 ± 0.5 | *0.222* | 4.4 ± 1.6  4.1 ± 1.3 | *0.252* | 4.8 ± 1.8  4.7 ± 1.7 | *0.164* |

*Key: *Dx Ix, diagnostic investigations; MDT, multidisciplinary team meetings; OPC, outpatient clinic appointments; SCC, squamous cell carcinoma; UC, undifferentiated carcinoma; AC, adenocarcinoma.*

**Supplementary File 9:** Diagnostic investigations performed in CCA diagnostic pathway.

| Diagnostic Investigation | Number of Patients (%) n=216 |
| --- | --- |
| Imaging  CT*  MRI (including MRCP)  Ultrasound  PET  Plain film x-ray  Nuclear bone scan  CT colonoscopy | 216 (100%)  130 (60.2%)  112 (51.9%)  41 (18.9%)  26 (12.1%)  2 (0.9%)  1 (0.5%) |
| Endoscopic Procedures  ERCP  Upper GI endoscopy  EUS  Colonoscopy  Cystoscopy  Hysteroscopy  Wireless endoscopy | 77 (35.6%)  36 (16.7%)  20 (9.3%)  15 (6.9%)  1 (0.5%)  1 (0.5%)  1 (0.5%) |

*Key: *CT, computed tomography; MRI, magnetic resonance imaging; MRCP, magnetic resonance cholangiopancreatography; PET, position emission tomography; ERCP, endoscopic retrograde cholangiopancreatography; GI, gastrointestinal; EUS, endoscopic ultrasound.*
